# A first trimester prediction model for large for gestational age infants: a preliminary study

**DOI:** 10.1186/s12884-021-04127-3

**Published:** 2021-09-24

**Authors:** Francesca Monari, Daniela Menichini, Ludovica Spano’ Bascio, Giovanni Grandi, Federico Banchelli, Isabella Neri, Roberto D’Amico, Fabio Facchinetti

**Affiliations:** 1 Obstetrics Unit, Mother Infant Department, University Hospital Policlinico of Modena, Modena, Italy; 2 International Doctorate School in Clinical and Experimental Medicine, Department of Biomedical, Metabolic and Neural Sciences, University of Modena and Reggio Emilia, Via del Pozzo 71, 41121 Modena, Italy; 3 Department of Diagnostic, Clinical and Public Health Medicine, Statistics Unit, University of Modena and Reggio Emilia, Modena, Italy

**Keywords:** Prediction model, LGA, First trimester, Biochemical markers, Biophysical markers

## Abstract

**Background:**

Large for gestational age infants (LGA) have increased risk of adverse short-term perinatal outcomes. This study aims to develop a multivariable prediction model for the risk of giving birth to a LGA baby, by using biochemical, biophysical, anamnestic, and clinical maternal characteristics available at first trimester.

**Methods:**

Prospective study that included all singleton pregnancies attending the first trimester aneuploidy screening at the Obstetric Unit of the University Hospital of Modena, in Northern Italy, between June 2018 and December 2019.

**Results:**

A total of 503 consecutive women were included in the analysis. The final prediction model for LGA, included multiparity (OR = 2.8, 95% CI: 1.6–4.9, *p* = 0.001), pre-pregnancy BMI (OR = 1.08, 95% CI: 1.03–1.14, *p* = 0.002) and PAPP-A MoM (OR = 1.43, 95% CI: 1.08–1.90, *p* = 0.013). The area under the ROC curve was 70.5%, indicating a satisfactory predictive accuracy. The best predictive cut-off for this score was equal to − 1.378, which corresponds to a 20.1% probability of having a LGA infant. By using such a cut-off, the risk of LGA can be predicted in our sample with sensitivity of 55.2% and specificity of 79.0%.

**Conclusion:**

At first trimester, a model including multiparity, pre-pregnancy BMI and PAPP-A satisfactorily predicted the risk of giving birth to a LGA infant. This promising tool, once applied early in pregnancy, would identify women deserving targeted interventions.

**Trial registration:**

ClinicalTrials.gov NCT04838431, 09/04/2021.

## Introduction

It is well recognized that large for gestational age infants (LGA), defined as a babies born with a birthweight above the 90th centile for gestational age and gender, have increased risks of adverse short-term perinatal outcomes i.e., induction of labor, instrumental vaginal delivery, caesarean section, shoulder dystocia, and perinatal asphyxia [1–4]. These neonates also face long-term increased risks of death, hospitalization as well as increased occurrence of obesity, hypertension and type 2 diabetes later in life [5, 6].

LGA is usually a result of maternal diabetes, obesity, and an excessive weight gain during pregnancy. However, there are several other factors that interplay with fetal growth as the genetics, intrauterine environment, nutrition, and placental function.

Among others, plasma protein A (PAPP-A), an enzyme produced by the placenta and by several maternal tissues [7] which releases insulin-like growth factor from its carrier protein, have been related with size at birth [8]. Indeed, increased PAPP-A between the 9–12 weeks was associated with LGA babies, in normal weight women [9]. Also β human chorionic gonadotropin (β-hCG), which stimulates trophoblast invasion, [10] has been involved since its concentrations correlated with both placental volume and birthweight [11, 12].

Fetal growth seems to be affected by other biochemical factors as placental growth factor (PlGF), an angiogenic molecule also produced by the placenta [13]. Higher PlGF levels are related with a better vascular function, which in turn increases glucose transport leading to higher glucose and nutrients exposure to the offspring. An association of PlGF levels with LGA babies, namely in women with preexisting diabetes, has been reported [14]. Furthermore, abnormal levels of inhibin-A were associated to adverse perinatal outcomes, also impacting on fetal growth [15].

Previous prediction models for LGA did not take into account the above reported factors [12] and have been developed either in obese mothers [16] or in women with gestational diabetes mellitus (GDM) [17, 18]. Although PAPP-A, free β-hCG, lipid [19] and inflammatory (interleukin-6, IL-6) [20] markers have all been included in those models, it is important to remember that GDM is only a proxy of LGA. Thus, the objective of our prospective study is to develop a multivariable prediction model for the risk of having a LGA infant, by using biochemical, biophysical, anamnestic, and clinical maternal characteristics, all available at first trimester.

## Materials and methods

Singleton pregnancies between June 2018 and December 2019 were included in the study among women attending the first trimester Down syndrome screening at the University Hospital of Modena, in the North of Italy (tertiary Hospital).

The study was approved by the Ethical Committee of the Area Vasta Emilia Nord (AVEN, protocol AOU: 0001395/20) and registered (ClinicalTrials.gov: NCT04838431, 09/04/2021). A written informed consent was obtained. Women were included if crown–rump length ranged 45–80 mm and no signs of miscarriage were present. Pregnancies with major fetal abnormalities were excluded from the study.

For each subject, blood sample was collected in fasting conditions, then centrifuged, and the serum stored at minus 80 °C, for subsequent biochemical analyses. PAPP-A, PlGF, free β-hCG and Inibin A have been collected following the indications of the preeclampsia screening proposed by the Fetal Medicine Foundation (FMF) [21] and were measured with the automated DELFIA EXPRESS system (Thermo Fisher Scientific, Perkin Elmer®). To evaluate the lipid profile, high density lipoproteins (HDL) and triglycerides (TG) were collected, in addiction to Insulin levels and Interleukin-6 as mediators of glucose tolerance and inflammation, respectively. These biochemical markers were all measured through routine laboratory methods.

### Assays performance

Precision of serum makers and cytokine measurements was determined by calculating the coefficient of variation (CV), at different concentrations. The intra-assay CV ranged 1.2–1.4 for PAPP-A, 2.0–2.4 for free β-hCG, 4.1–2.1 for PlGF and 7.2–2.1 for Inhibin A. The inter-assay CV ranged 2.1–2.5 for PAPP-A, 1.4–2.6 for free β-hCG, 1.5–3.0 for PlGF, 2.3–2.0 for Inhibin A and 1.5–5.0 for IL-6.

The Limit of Detection (LOD) for the serum makers and cytokine measurements was as following PAPP-A < 5 mU/L, free β-hCG < 0.2 ng/mL, PlGF < 1.9 pg/mL, Inhibin A = 5,7 pg/mL and IL-6 = 1.5 pg/mL.

The Limit of Quantitation (LOQ) was < 15 mU/L for PAPP-A, < 0.3 ng/mL for free β-hCG, 3.3 pg/mL for PlGF, 6.3 pg/mL for Inhibin A and 5 pg/mL for IL-6.

The mean arterial pressure (MAP) was measured with validated automated devices (Dinamap, BLTV6XX). After the women were seated and allowed to rest for 3–5 min, normal (22 to 32 cm) adult cuffs were fitted to their both arms. This was repeated two times with 1 min break in between. The MAP was calculated with the formula MAP = DBP + 1/3(SBP – DBP) [22], where DBP represents diastolic blood pressure and SBP, systolic blood pressure. We calculated the final MAP as the average of all four measurements. Uterine artery Doppler studies including pulsatility index were measured through trans-abdominal ultrasound (Voluson E8 or Voluson E10) examinations. As indicated in FMF, when carrying out Doppler studies, color flow mapping was used to identify each uterine artery along the side of the cervix and uterus at the level of internal os. Pulsed wave Doppler imaging was used with the sampling gate set at 2 mm to cover the whole vessel, and care was taken to ensure that the angle of insonation was less than 30°. When three to five similar consecutive waveforms had been obtained, PI was measured. The uterine artery PI was calculated by adding the right and left pulsatility index together, divided by two. All ultrasound and Doppler studies were carried out by a physician who had received the appropriate certificate of competence in the 11–13 + 6 week scans and Doppler study from the FMF [23].

Data on pregnancy outcome were collected from the hospital maternity records or directly from women if delivered elsewhere.

Data on pregnancy outcome such as weight gain according to the Institute of Medicine (IOM) recommendations, gestational diabetes, stillbirth, abruptio placentae, gestational hypertension and preterm delivery were collected from the hospital maternity records or directly from patients if delivered in another setting. Medical records were reviewed by research associates to obtain anonymized data on mothers and their newborns. Maternal medical history included family history of medical conditions, chronic hypertension, or pre-existing diabetes, defined as occurring before pregnancy or within the first trimester. Neonatal outcomes included birthweight, gender, Apgar scores, admission to the neonatal intensive care unit (NICU), length of stay, neonatal morbidities, and mortality. Neonatal anthropometric measures were collected to define newborns as small, appropriate, or large for gestational age (SGA, AGA, LGA, respectively) according to the Italian curves for neonatal growth validated by Bertino E. et al. 2010 [24]. These curves/charts consider not only the birthweight (BW), but also the body length (BL), and head circumference (HC), sex, and birth order. Therefore, neonates whose BW, BL and HC values fell above the 90th centile were considered LGA. All data were organized in a password protected database.

### Statistical analysis

Quantitative variables were described as the mean ± standard deviation (SD), whereas qualitative variables were described as the absolute and percentage frequencies. The multivariable prediction model was developed by carrying out the following steps. Firstly, univariate logistic regression models were used to assess the relationship among each relevant independent variable and the risk of having a LGA infant. During this step, several alternative parameterizations were used for quantitative variables, including linear effect; step effect based on median or first / third quartile; step effect based on clinically meaningful values; linear effect on multiples of median (MoM). The metabolic syndrome was defined as the presence of at least three of the following variables: HDL < 50 mg/dl, TG ≥ 150 mg/dl, SBP ≥ 130 mmHg, DBP ≥ 85 mmHg, BMI ≥ 30 kg/m2 [25].

The variables that were associated to LGA risk with *p*-value < 0.10 in the univariate analyses were considered for inclusion in a multivariable logistic model. The final prediction model was determined by a stepwise backward selection procedure in which only independent variables associated to LGA risk with p-value < 0.05 were retained. Results of logistic models were reported as the Odds Ratio (OR) with 95% confidence interval and Wald p-value. The overall accuracy of the estimated prediction model was assessed by using the area under the ROC curve with 95% confidence interval. The formula for the predictive score for LGA was equal to the linear predictor of the final model, in which each independent variable was weighted proportionally to its log OR. The predicted probability of having a LGA infant can be calculated as exp.(score) / [1 + exp.(score)]. Furthermore, we calculated the best score threshold by using the Youden’s rule and we reported the associated values for sensitivity and specificity. Statistical analyses were performed by using R 3.6.3 software (The R Foundation for Statistical Computing, Wien).

## Results

Five-hundred and fifteen women agreed to participate in this prospective study. Of them, 2 had spontaneous miscarriage in the second trimester, 2 underwent a therapeutic termination of pregnancy (one for trisomy 21 and one for fetal congenital heart disease detected at ultrasound) while 8 women not delivering at our center were lost to follow-up. Therefore, a total of 503 women were included in the final analysis.

The maternal baseline characteristics were compared between those giving birth to a LGA neonate was (87, 17.3%) and the remnants (416) delivering normal weight infants (Table 1).

**Table 1:** Maternal baseline characteristics

	Non LGA (***N*** = 416)	LGA (***N*** = 87)	***P*** value
**Maternal age** (mean ± SD)	32.4 ± 4.5	33.0 ± 4.8	0.28
**Low education level** (≤ 8 years)	52 (12.5)	13 (14.9)	0.44
**Italian place of origin**	363 (87.2)	11 (87.3)	0.12
**Smoking habits**	27 (6.4)	4 (4.6)	0.54
**BMI classes**			**0.000**
Underweight	19 (4.5)	0	
Normal weight	266 (63.9)	36 (41.4)*	
Overweight	67 (16.1)	29 (33.3)	
Obese	56 (13.5)	19 (21.8)	
Morbidly Obese	8 (1.9)	3 (3.5)	
**Nulliparity**	263 (63.2)	36 (41.4)*	**0.0001**
**Assisted reproductive conception**	15 (3.7)	2 (2.3)	0.73
**Preexisting Diabetes Mellitus**	3 (0.7)	3 (3.4)*	**0.03**
**Chronic Hypertension**	15 (3.5)	3 (3.4)	0.97
**Metabolic Syndrome**^a^	21 (5.0)	7 (8.0)	0.23

Data are reported as numbers with percentage in brackets

* p value < 0.05

^a^ Metabolic syndrome is defined as the presence of at least 3 of the 5 following variables:

- HDL < 50 mg/dl

- TG >/= 150 mg/dl

- SBP >/= 130 mmHg

- DBP >/= 85 mmHg

- BMI >/= 30 kg/m^2^

The two groups were similar for maternal age and education level, while the rate of women with Italian place of origin was lower in the LGA group. Moreover, the LGA group included less normal weight while more multiparous women. A higher rate of women with preexisting diabetes mellitus was also found in the LGA group, while metabolic syndrome was similarly represented in the two groups.

Table 2 summarizes the biochemical and biophysical markers for LGA at first trimester enrollment.

**Table 2:** Biochemical and biophysical markers under evaluation

	Non LGA (***N*** = 416)	LGA (N = 87)	P value
**MAP > 90 mmHg**	116 (27.1)	32 (36.8)	0.06
**Uterine Doppler PI > 90th centile**	44 (10.3)	7 (8.0)	0.51
**Insulin** (μUI/mL)	11.7 ± 1.47	15.0 ± 4.35	0.09
**Triglycerides** (mg/dL)	107.32 ± 4.21	116.00 ± 9.94	0.09
**HDL** (mg/dL)	64.38 ± 1.1	62.48 ± 2.5	0.16
**Inhibin A** (pg/mL)	322.13 ± 16.6	342.92 ± 46.8	0.33
**Interleukin-6** (pg/mL)	1151.05 ± 191.6	986.18 ± 391.7	0.47
**PAPP-A** (MoM)	1.40 ± 0.75	1.53 ± 0.86*	**0.04**
**free β-hCG** (MoM)	1.12 ± 0.60	1.01 ± 0.58	0.36
**PlGF** (MoM)	1.23 ± 0.50	1.28 ± 0.55	0.11
**Fetal cardiac frequency > 162 bpm**	191 (44.6)	31 (35.6)	0.10

Mean values ± SD and numbers with percentage in brackets are reported

* *p* value < 0.05

MAP: mean arterial pressure; MoM: Multiple of the median

Mean arterial pressure > 90 mmHg and the mean pulsatility index of the uterine artery doppler >90th centile, were similar between the two groups as well as plasma insulin, triglycerides, and HDL. Placental and vascular markers as PlGF, inhibin A and IL-6 mean values were comparable while the MoM of PAPP-A significantly differed between groups.

Pregnancy outcomes are reported in Table 3. No significant differences were detected as far as GDM, pregnancy induced hypertension (PIH) or preeclampsia (PE). Interestingly, the number of women who gained more weight than recommended by the Institute of Medicine (IOM) (47.1% vs 20.9%) was increased in LGA group (Table 3).

**Table 3:** Pregnancy Outcomes

	Non LGA (***N*** = 416)	LGA (N = 87)	P value
**GDM**			0.15
Dietary treatment	46 (10.7%)	12 (13.7%)	
Insulin treatment	11 (2.6%)	7 (8.0%)	
**Pregnancy-induced Hypertension**	23 (5.3%)	4 (4.6%)	0.73
**Pre-eclampsia**	6 (1.4%)	1 (1.1%)	0.83
**Weight gain above IOM recommendations**	87 (20.9%)	41 (47.1%)	**0.0000**
**Abruptio Placentae**	3 (0.7%)	1 (1.1%)	0.68
**Fetal Growth Restriction**	6 (0.7%)	0 (0.0%)	0.26

Table 4 shows the main perinatal outcomes. While a significantly higher percentage of women with a LGA baby underwent induction of labor, the rate of cesarean section and vaginal operative deliveries was similar between the two groups. Neonatal adverse outcomes, as NICU admission, acidosis at birth, defined as an umbilical artery pH < 7.2 or Apgar score < 7 at 5th minute were comparable.

**Table 4:** Perinatal Outcomes

	Non LGA (***N*** = 428)	LGA (N = 87)	P value
**Mode of Labour**			**0.01**
Spontaneous	303 (70.8%)	52 (59.7%)*	
Induced	101 (23.6%)	35 (40.2%)*	
**Delivery**			0.10
Vaginal	302 (70.6%)	61 (70.1%)	
Vaginal Operative	28 (6.5%)	3 (3.4%)	
Cesarean Section	98 (22.9%)	23 (26.4%)	
**Male gender**	214 (50.0%)	49 (56.3%)	0.14
**NICU admission**	14 (3.3%)	1 (1.1%)	0.14
**Umbilical a. pH < 7.2**	23 (5.4%)	6 (3.4%)	0.43
**5th min. Apgar < 7**	6 (1.4%)	1 (1.1%)	0.89

* p value < 0.05

### Early prediction model of LGA risk

Based on the parameters available in at first trimester, a backward stepwise logistic regression was performed to identify potential predictors of LGA among 14 relevant independent variables (age, parity, Italian place of origin, pre-pregnancy BMI, preexisting diabetes mellitus, HDL, TG, insulin, PAPP-A, PlGF, IL-6, inhibin A, fetal cardiac frequency and metabolic syndrome). The results of both univariate and multivariable analyses were reported in Table 5. At univariate analysis LGA babies were associated with multiparity (OR = 2.41, 95%CI 1.51–3.86, *p* = 0.001), pre-pregnancy BMI (OR = 1.08, 95%CI 1.04–1.12, p = 0.001), pre-existing diabetes (OR = 5.04, 95%CI 1.00–25.38, *p* = 0.050) and PAPP-A MoM (OR = 1.30, 95%CI 1.00–1.70, *p* = 0.051).

**Table 5:** Development of the prediction model for LGA risk

	Univariate analyses (***n*** = 503)				Multivariable prediction model (***n*** = 434)			
	OR	95% CI		p	OR	95% CI		p
**Maternal Age** (+ 1 year)	1.03	0.98	1.08	0.283				
**Multiparity**	**2.41**	**1.51**	**3.86**	**0.001**	**2.80**	**1.61**	**4.87**	**0.001**
**Italian place of origin**	1.17	0.61	2.45	0.065				
**Pre-pregnancy BMI** (+ 1 kg/m^2^)	**1.08**	**1.04**	**1.12**	**0.001**	**1.08**	**1.03**	**1.14**	**0.002**
**Pre-existing diabetes**	**5.04**	**1.00**	**25.38**	**0.050**				
**HDL ≥ 50 mg/dL**	0.63	0.30	1.29	0.206				
**TG ≥ 150 mg/dL**	1.76	0.96	3.23	0.068				
**Insulin ≥ 24** (+ 1μUI/mL)	1.74	0.92	3.29	0.091				
**PAPP-A** (+ 1 MoM)	**1.30**	**1.00**	**1.70**	**0.051**	**1.43**	**1.08**	**1.90**	**0.013**
**PlGF** (+ 1 MoM)	1.21	0.75	1.97	0.432				
**IL-6** (+ 1 pg/mL)	1.00	1.00	1.00	0.477				
**Inhibin A** (+ 1 pg/mL)	1.00	1.00	1.00	0.338				
**FCF ≥ 162**	0.69	0.41	1.16	0.157				
**Metabolic Syndrome**	1.70	0.70	4.12	0.244				

The variables that were associated to LGA risk with p-value < 0.10 in the univariate analyses were considered for inclusion in a multivariable logistic model. The final prediction model was determined by a stepwise backward selection procedure in which only independent variables associated to LGA risk with *p*-value < 0.05 were retained

The final prediction model for LGA at multivariable analysis included the following independent variables: multiparity (OR = 2.8, 95% CI = 1.6–4.9, p = 0.001), pre-pregnancy BMI (OR = 1.08, 95%CI 1.03–1.14, *p* = 0.002) and PAPP-A MoM (OR = 1.43, 95%CI 1.08–1.90, *p* = 0.013) (Table 5).

There was no significant effect modification on the risk of LGA among the three variables that were selected for our final predictive model (all, *p* > 0.05).

**Fig. 1:**
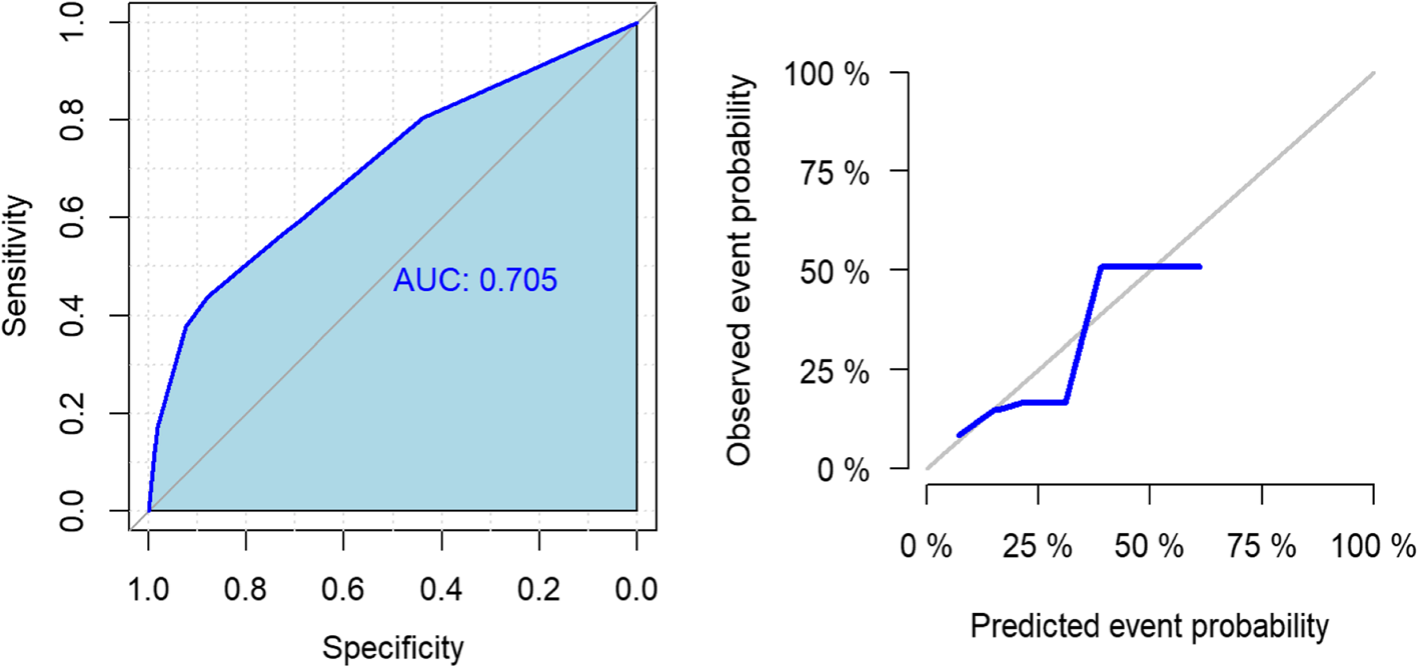
ROC curve and predicted versus observed event probability plot

The area under the ROC curve was 70.5%, indicating a moderate predictive accuracy (Fig. 1).

The prediction score for LGA risk was as follows: Score = − 4.565 + 1.030 * multiparous + 0.079 * BMI + 0.358 * PAPP-A MoM. The best predictive cut-off for this score was equal to − 1.378, which corresponds to a 20.1% probability of having a LGA infant. By using such a cut-off, the risk of LGA can be predicted in our sample with sensitivity of 55.2% and specificity of 79.0%

## Discussion

This prospective study developed a tool for the early pregnancy prediction of LGA infants in a non-selected population. Previous prediction models for LGA and macrosomia have been build-up in larger, although selected populations, i.e. within obese subjects [16] or in women with a diagnosis of GDM [17, 18]. Moreover, despite several studies developed predictive models of macrosomal fetus through amniotic fluid [26] and ultrasounds [27], they were performed later, at mid pregnancy. Only one study used first-trimester markers for macrosomia reporting the prediction of macrosomia by fetal NT, free β-hCG and PAPP-A [28]. Other studies, conducted later in pregnancy found that gestational weight gain [18] and early third-trimester sonographic fetal biometry are predictive of LGA infant at term [17]. Moreover, levels of PAPP-A and free β-hCG were significantly higher in obese women with an LGA infant compared with obese women with normal-weight infants [16]. On the contrary, in our small series, we included all classes of pre-pregnancy BMI, women with different ethnicity, parity and with heterogenous obstetric history.

This study thus confirms and enlarges the observation that there is a linear association between MoM of PAPP-A levels and LGA, as firstly reported in a cohort of GDM women [29] and later found also in pre-pregnancy obese mothers [16]. This finding is compatible with the observations that glucose levels affect placentation by influencing trophoblast invasion [30]. Indeed, low levels of PAPP-A were reported to be associated with poor early placentation resulting in perinatal complications such as fetal growth restriction, fetal demise, preterm birth, and pre-eclampsia [31].

Moreover, we confirmed that either multiparity and maternal pre-pregnancy BMI are good predictors of increased birthweight and LGA infants [32–34], possibly due to the faster fetal growth transfer in those conditions [35]. On the other hand, it is now assessed that the levels of placental growth factor in maternal blood, as well as the measures of uterine artery pulsatility index, should be excluded among possible markers for LGA [12]. Despite promising, also the other new markers we tested, Inhibin A and Interleukin 6 did not enter in the equation.

The early pregnancy prediction model we obtained is mathematically worth of note, with a satisfactory specificity and an AUC of 0.705. This tool, in line with the concept of “Inverted Pyramid of Care” [36], adds LGA among those pregnancy complications that could be predicted, hence prevented through timely interventions. Furthermore, our predictive model has the advantage of using easily and early available variables as they are biophysical (pre-pregnancy BMI), anamnestic (multiparity) or plasma markers (i.e. PAPP-A), unlike those previously mentioned, such as invasive investigations on amniotic fluid [26] or ultrasounds which become accurate only later in pregnancy.

Several studies focused the attention on the excessive gestational weight gain which is a well-recognized factor contributing to LGA [37]. Moreover, also interpregnancy weight increase has been found to be associated with LGA [38]. Implementing an early customized low glycemic index, low fat diet together with the stimulation of physical activity has the potential to reduce the risk of LGA baby in some populations [39, 40]. Unfortunately, quality, timing and adherence to intervention are factors significantly affecting success [39]. In many studies, the window for intervention was too narrow to effectively change women lifestyle and/or it could be too late since the fetal metabolic “programming” was already set [41]. Hence, timing of intervention seems crucial to prevents disorders, and the model we developed helps to start very early in pregnancy.

In summary, multiparity, increased maternal pre-pregnancy BMI and high PAPP-A levels measured at first trimester predict the risk of having a LGA infant, with a good specificity. This helps identify the population which deserves early interventions. We hope this formula will undergo validation in further, larger populations.

## Data Availability

The datasets used and/or analyzed during the current study are available from the corresponding author on reasonable request.
